# Human Skin-Inspired Staggered Microstructures for Optimizing Sensitivity of Flexible Pressure Sensor

**DOI:** 10.3390/s25082415

**Published:** 2025-04-11

**Authors:** Kechen Li, Yuanyuan Yang

**Affiliations:** School of Aerospace Engineering, Xiamen University, Xiamen 361000, China; 35120231151732@stu.xmu.edu.cn

**Keywords:** flexible sensor, pressure sensing, staggered microstructure, human skin-inspired, gesture recognition, vital sign monitoring

## Abstract

Flexible pressure sensors play a significant role in wearable electronics, human–machine interfaces, and health monitoring, and improving their performance has always been a major focus of research. Various microstructures have been proposed to enhance sensitivity, particularly when tilted. However, unidirectional tilting may create a shift in contact surfaces, reducing accuracy in pressure detection. To address these limitations, this study introduces a capacitive pressure sensor with a staggered tilted column microstructure, inspired by the elaborate network of epidermis and dermis layers within human skin. The simulation and experiment results reveal that the developed sensor has high sensitivity and responds rapidly to applied forces, making it suitable for real-time applications. Demonstrations of gesture recognition and physiological monitoring highlight its practical potential. These findings underscore the effectiveness of the staggered microstructure in improving sensor performance and its applicability in next-generation flexible sensors.

## 1. Introduction

Flexible pressure sensors are significant in wearable electronics, soft robotics, and human–machine interfaces [1,2,3,4,5,6,7]. These sensors are valued for their ability to conform to complex surfaces and accurately measure mechanical stimuli. Further, these sensors can be broadly classified based on their sensing mechanisms, including resistive, capacitive, and triboelectric sensors [8,9,10,11]. Among these, capacitive pressure sensors have garnered much attention for their simple fabrication process, rapid response, and low hysteresis. Achieving high sensitivity in flexible capacitive sensors remains a key area of exploration for many researchers. It is critical as it determines the sensor’s ability to detect small changes in pressure, which is essential for applications that require precision. To meet these demands, researchers have been actively investigating various methods to enhance sensor sensitivity, thereby aiming to develop sensors that are both highly responsive and reliable under diverse conditions.

To enhance sensor sensitivity, one of the most effective strategies has been the design of microstructures within the sensor. These microstructures—such as pyramids, domes, and other geometric patterns—function by increasing the effective surface area and compressibility, thereby enhancing the sensor’s responsiveness [12,13,14,15,16,17]. Among these designs, tilted structures—where the microstructures are angled rather than perpendicular—have demonstrated superior sensitivity due to their increased compressibility under pressure [18,19,20]. However, when these microstructures are all tilted in a single direction, certain limitations can arise. Specifically, a unidirectional tilt can result in a shift in the contact surface, impairing the sensor’s ability to detect pressure accurately, thus reducing overall sensitivity.

**Figure 1:**
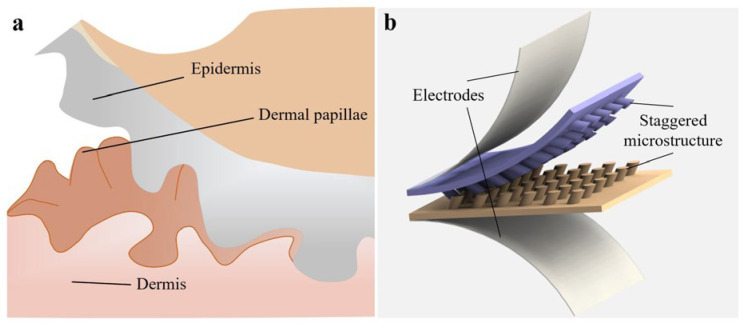
Sensor design with human skin-inspired staggered microstructures. (**a**) The elaborate network within the epidermis and dermis layers allows the skin to effectively distribute stress, thereby ensuring high sensitivity across a wide range of applied forces. (**b**) The sensor features a dielectric layer composed of staggered tilted columns, where the tilt direction of each row of columns alternates.

Inspiration to overcome these limitations can be drawn from the complex, multidirectional, and staggered structure of human skin, particularly the intricate tissue found between the epidermis and dermis (Figure 1a). Skin serves not only as a protective barrier against environmental hazards but also plays a crucial role in sensing and transmitting external stimuli, converting them into physiological signals. The elaborate network within the epidermis and dermis layers allows the skin to effectively distribute stress, thus ensuring high sensitivity across a wide range of applied forces. This naturally evolved microstructure provides valuable insights for designing flexible pressure sensors that have enhanced performance, thereby providing a model for achieving greater sensitivity and reliability in sensor applications. Such a model has been referred to in certain studies in which the sensors are designed using interleaved structures [21,22,23]. While such structures demonstrate improved performance over conventional designs, their microstructural morphologies remain largely uniform and unidirectional. To better mimic the multidirectional architecture of natural skin tissue, further development of heterogeneous microstructure designs with optimized arrangements should be explored.

In this study, we present a flexible capacitive pressure sensor inspired by the staggered, multidirectional microstructure of human skin. The sensor features a dielectric layer composed of staggered tilted columns, where the tilt direction of each row of columns alternates (Figure 1b). This biomimetic design emulates the natural architecture of skin tissue, thus promoting enhanced stress distribution and significantly improving sensitivity. According to the experiment results, the sensor exhibits superior sensitivity compared to conventional unidirectional designs. Demonstrations of the applications of this sensor in gesture recognition and physiological monitoring are also presented. The findings suggest that this biomimetic approach provides a promising strategy for advancing the performance of flexible pressure sensors.

## 2. Materials and Methods

### 2.1. Fabrication of the Sensor

**Figure 2:**
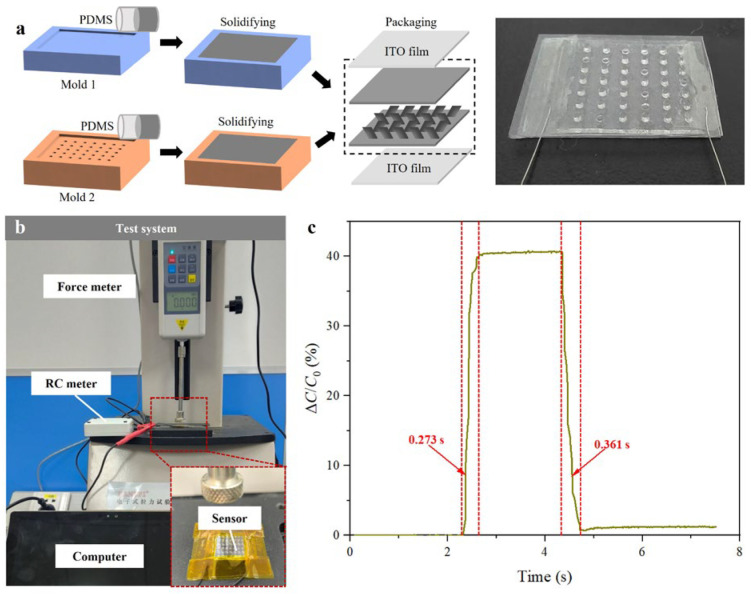
Fabrication and characterization of the sensor. (**a**) The pressure sensor is fabricated and assembled by layering ITO film, PDMS dielectric layers, and another ITO film from the bottom to the top. (**b**) The system setup for pressure and capacitance characterization consists of a force meter (HP-20, HANDPI, Yueqing, China) and a resistance–capacitance (RC) meter (LZ-01ARC, Shanghai, China). (**c**) The sensor responds for approximately 0.2–0.3 s, thereby demonstrating its capability to accurately detect and transmit pressure changes with minimal delay.

The fabrication of microstructures requires a molding technique, and the molds are fabricated using a Donzy Reflect2 light-curing 3D printer (DONZY, Shenzhen, China, printing accuracy: 0.1 mm). First, polymethylhydrosiloxane (PDMS) is prepared in a 10:1 weight ratio of base to curing agent and stirred for 10 min. Thereafter, it is vacuumed for 30 min to remove air bubbles. The PDMS is poured into molds 1 (no pattern) and 2 (pattern with microstructures) and cured at 80 °C (Figure 2a), yielding dielectric layers with the designed microstructures. The geometric parameters of the interlaced microstructures are designed by considering sensitivity enhancement (via increased compressibility and contact area) and fabrication feasibility (ensuring successful demolding and structural integrity). Thereafter, the dimensions (2.5 mm spacing, 500 μm height, 1 mm diameter, and 45° tilt angle) are selected for the microstructures. Then, the pressure sensor is assembled by layering it with an indium–tin–oxide (ITO) film (thickness: 23 nm, resistance: 100 Ω), PDMS dielectric layers (thickness: 800 μm), and another ITO film from bottom to top. Electrical wires are attached to the ITO layers for signal output. Finally, the sensing unit is packaged using plasma treatment on the connecting surfaces.

### 2.2. Characterization of the Sensor

The microstructures of the dielectric PDMS are characterized using a digital microscope (DINO AM73915MZT, VIDY OPTICS, Wuxi, China), including straight cylinders, unidirectionally tilted cylinders, and staggered tilted cylinders. The base diameter of these cylinders is 1 mm, and the height is 500 μm. For the tilted cylinders, the slant angle is 45°. The 45-degree angle is selected to ensure the molding rate of the dielectric layer while allowing the sensor to have the highest possible sensitivity.

The system setup for pressure and capacitance characterization consists of a force meter (HP-20) and a resistance–capacitance (RC) meter (LZ-01ARC). During the experiment, the applied force and the capacitance variation between the two layers are recorded.

### 2.3. Static Stress Simulation of the Sensor

Finite element models of sensors with three microstructure designs—straight, unidirectionally tilted, and staggered tilted configurations—are developed in SolidWorks. The models are parametrically designed to match the material properties of the actual sensor. In the simulation setup, the bottom electrode is fixed as a boundary condition, while uniformly distributed loads ranging from 0 kPa to 10 kPa are applied normally to the top electrode surface to evaluate the mechanical response.

## 3. Results

### 3.1. Sensing Response

The fabricated sensor is constructed using ITO electrode films and a sandwiched PDMS dielectric layer, which contains staggered microstructures similar to the dermal papillae in human skin. The dermal papillae feature an interlocking and staggered structure, which enables enhanced mechanical interaction and optimized stress distribution. The staggered structure in the sensor mimics the layout of dermal papillae to enhance pressure sensitivity and ensure a reliable sensing performance. Further, the flexible pressure sensor is calibrated to evaluate its performance characteristics. The response time is tested by applying a force meter to the sensor and recording the output capacitance signal, the testing system of which is exhibited in Figure 2b. As depicted in Figure 2c, the sensor exhibits a fast response time, which is a critical parameter for applications that require real-time pressure detection. The sensor responds in approximately 0.2–0.3 s, thereby demonstrating its capability to accurately detect and transmit pressure changes with minimal delay. This rapid responsiveness is attributed to the optimized design of the dielectric layer, which enables efficient stress transfer and signal generation.

### 3.2. Mechanical Analysis

**Figure 3:**
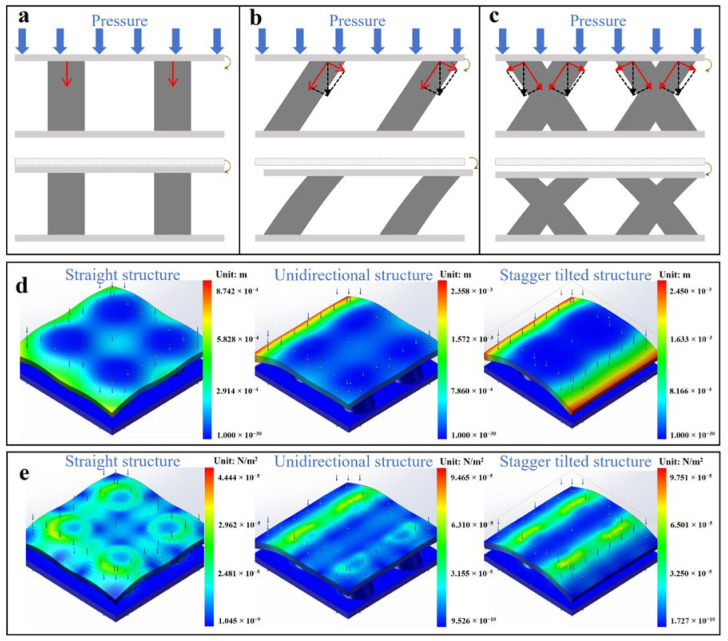
Mechanical analysis and simulation of sensors with different microstructures. (**a**) Mechanical analysis of the straight, (**b**) unidirectionally tilted, and (**c**) staggered tilted microstructures. (**d**) Simulation results of the upper capacitor’s displacement and (**e**) strain when pressure of 10 kPa is applied to the sensors.

To assess the advantages of the staggered tilted column microstructures, a comparative analysis is performed between the proposed sensor and sensors that feature unidirectional microstructures, as depicted in Figure 3. Compared with the straight design (Figure 3a), the tilted microstructures offer distinct advantages in sensitivity as they provide greater compressibility under pressure. This increased compressibility arises from the angled orientation, which enables the microstructures to undergo more substantial deformation due to the generation of a tangential force on the microstructures. Consequently, sensors with tilted microstructures typically exhibit higher sensitivity as they can better respond to applied pressure. Building on this, the staggered tilted column design further enhances sensitivity compared to a unidirectionally tilted one. In a unidirectionally tilted structure, all columns are angled in the same direction (Figure 3b). While this configuration improves the sensor’s sensitivity relative to straight microstructures, it introduces the possibility of a shift in the sensor’s surface, potentially compromising the sensor’s ability to accurately detect pressure and, thus, reducing its overall sensitivity. The plate displacement caused by the unidirectionally tilted structure reduces the pre-designed plate area, thus reducing the ability of the sensor to perceive pressure. In contrast, the staggered arrangement of tilted columns, where each row alternates in terms of tilt direction, mitigates these issues (Figure 3c). Compared with the straight design, both the unidirectionally tilted structure and the staggered tilted structure have greater deformability, but the staggered structure has more uniform stress distribution, more stable deformation, and can avoid the reduction in the plate area as compared to the unidirectionally tilted structure.

To further validate the aforementioned analysis, SolidWorks software (2025 SP 0.0) is employed to construct mechanical models, which represent three distinct microstructures, and perform a static stress analysis. Within the simulation environment, a uniformly distributed positive pressure of 10 kPa is applied to the upper plate of the sensors. The results depicting displacement variations in the capacitor model under stress are presented in Figure 3d. The maximum displacement of these three models under pressures ranging from 0 kPa to 10 kPa is presented in Figure S1. The findings indicate that with a tilted structure, the displacement of the upper plate is an order of magnitude greater than that observed with a straight structure; furthermore, displacement distribution within the staggered tilted structure is more uniform compared to that of its unidirectional counterpart, which is consistent with the analytical results. Moreover, a shift in the sensors’ surfaces is observed in sensors with unidirectionally tilted microstructures, whereas the staggered tilted microstructure configuration maintains structural stability (Figure S2). As revealed by the strain simulation results presented in Figure 3e, the strain values for tilted structures are almost double those recorded for straight structures. Similarly, strains associated with staggered tilted structures demonstrate greater performance when compared to those associated with unidirectionally tilted configurations. Such staggered tilted structures not only maximize compressibility but also enhance the sensor’s ability to maintain high sensitivity and accurate pressure detection across its entire surface.

### 3.3. Experimental Comparison

**Figure 4:**
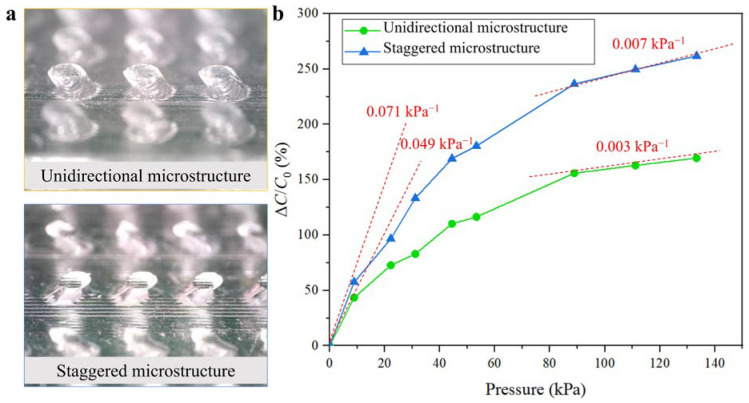
Experimental comparison of the sensors with different microstructures. (**a**) The microscope images of two microstructures. (**b**) The sensor with the staggered tilted column design exhibits a significantly higher sensitivity compared to the unidirectional sensor.

Experiments are performed to confirm the theoretical advantages of the staggered microstructure. For comparison, two kinds of microstructures are fabricated, as depicted in Figure 4a. Sensors constructed using these two dielectric layers are subjected to identical testing conditions, with incremental pressure applied to evaluate their sensitivity and overall performance. The sensitivity is quantified by evaluating the change in capacitance per unit of applied pressure. As depicted in Figure 4b, the results reveal that the sensor with the staggered tilted column design exhibits a significantly higher sensitivity compared to the unidirectional sensor. The staggered sensor consistently demonstrates superior performance across a wide range of pressures. Specifically, at low pressure levels (0–20 kPa), the staggered structure sensor has a sensitivity of 0.071 kpa^−1^, which is approximately 45% higher than that of the unidirectional sensor. At high pressure levels (90 kPa–130 kPa), the staggered structure sensor sensitivity of 0.007 kpa^−1^ is 133% higher. Overall, the results indicate that the staggered microstructure design could effectively improve the sensor’s sensitivity. Table S1 presents the comparison of sensitivity and response time between our sensor and a few related flexible pressure sensors with other microstructures. As exhibited, our sensor exhibits competitive sensitivity and response characteristics, further highlighting the advantages of the bio-inspired staggered microstructure design. Tests are also conducted to evaluate the hysteresis characteristics of the sensor. As shown in Figure S3, the sensor is subjected to increasing pressure from 0 kPa to 20 kPa, and then the external pressure is gradually withdrawn until it reaches 0 kPa. The result displays a narrow hysteresis window, thereby demonstrating the sensor’s reliable performance under dynamic pressure conditions. In addition, the durability of the sensor is tested, and the results are displayed in Figure S4. After 70 cycles, the sensor still maintains a good response consistency, thus indicating that the sensor has good reliability. These findings validate the effectiveness of the biomimetic design inspired by the multidirectional structure of human skin and suggest that this approach offers a significant advantage over conventional unidirectional designs.

### 3.4. Gesture Recognition Application

**Figure 5:**
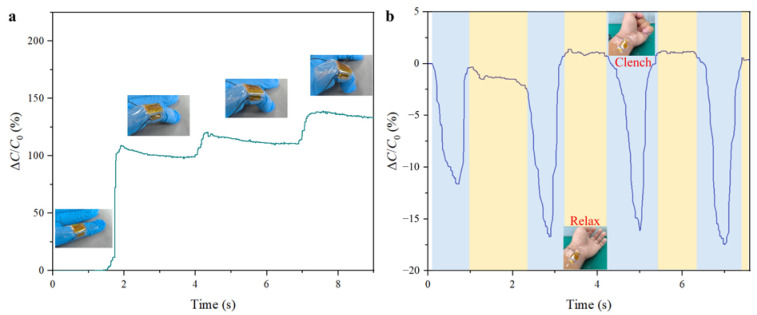
Gesture recognition applications of the sensor. (**a**) The sensor is placed on the joint of the finger to monitor and recognize various finger gestures. (**b**) The sensor could also be applied to the wrist to detect the clenching and unclenching of a fist.

The exceptional sensitivity and rapid response time of such a flexible pressure sensor make it an ideal candidate for advanced gesture recognition systems. To demonstrate its capabilities, two prototypes are developed that showcase how the sensor can be effectively used in real-time gesture detection. As shown in Figure 5a, the sensor is fixed on the knuckle of the hand to detect and identify finger bending movements. The staggered microstructure design enables the sensor to detect the changes in stress as the fingers bend and also facilitates precise differentiation among varying degrees of finger flexion, thereby providing a high-resolution output that accurately captures each gesture. This characteristic could be further applied in wearable devices aimed for use in human–computer interaction, where precise finger movements need to be translated into commands or inputs. The sensor could also be applied to the wrist to detect the clenching and unclenching of a fist as well. However, the complex biomechanics of the wrist, involving multiple tendons and muscles, present a challenge for accurate pressure sensing. As depicted in Figure 5b, the sensor’s enhanced sensitivity ensures that it can reliably monitor the variations in pressure across the wrist as the fist transitions between an open and closed state. This enables the sensor to differentiate between subtle variations in the intensity of the clenching motion, thus offering nuanced data that could be used in gesture-based control systems. Such an application could be extended to various fields, including gaming, robotics, and wearable health monitoring devices, where the precise detection of hand movements is critical.

### 3.5. Physiological Monitoring Application

**Figure 6:**
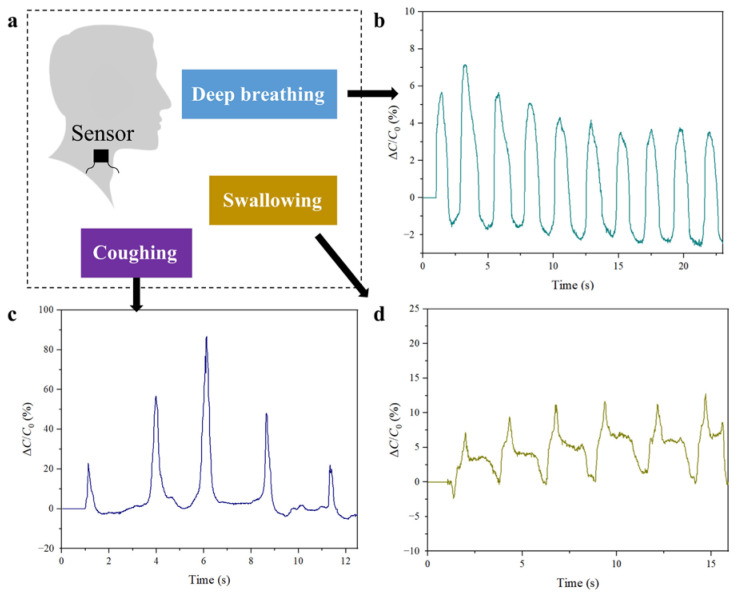
Physiological monitoring applications of the sensor. (**a**) The sensor is applied to the neck, where activities such as deep breathing, coughing, and swallowing generate distinct pressure patterns. (**b**) The sensor was able to capture the rhythmic pressure changes associated with deep breathing. (**c**) The sensor was able to detect the sharp pressure spikes characteristic of coughing, with larger capacitance variations compared to deep breathing. (**d**) The sensor could effectively monitor swallowing activity, with a longer duration of capacitance variation and a characteristic initial spike followed by a more gradual change, thereby mirroring the swallowing process.

Apart from gesture recognition, the sensor’s ability to detect subtle pressure variations holds great promise for health monitoring, particularly in the continuous and noninvasive detection of physiological activities. This potential is demonstrated by applying the sensor to the neck, where activities such as deep breathing, coughing, and swallowing generate distinct pressure patterns (Figure 6a). As illustrated in Figure 6b, the sensor’s high sensitivity enables it to accurately capture the rhythmic pressure changes associated with deep breathing. In Figure 6c, the sensor precisely detects the sharp pressure spikes characteristic of five coughs with different intensities within 12 s, with larger capacitance variations compared to deep breathing, thus reflecting the distinct nature of these activities. Additionally, the sensor effectively monitors swallowing activity, as illustrated in Figure 6d. Owing to the high sensitivity and prompt response of the sensor, the swallowing process is consistently detected, with a longer duration of capacitance variation and a characteristic initial spike followed by a more gradual change. Overall, the sensor’s versatility and precision in monitoring these physiological activities emphasize its potential as a multifunctional component in next-generation wearable technologies.

## 4. Discussion

In this study, we introduced a flexible capacitive pressure sensor inspired by the staggered, multidirectional microstructure of human skin. The experimental results demonstrated that the staggered tilted column microstructure significantly enhances the sensitivity of flexible pressure sensors when compared to conventional unidirectional designs. By emulating the multidirectional tissue structure inherent in human skin, this design achieved a more uniform stress distribution across the sensor’s surface, thus effectively minimizing issues related to shifting contact surfaces and enhancing overall accuracy in pressure detection. These findings provide a novel perspective by emphasizing the benefits of a staggered, multidirectional approach. The improved sensitivity achieved with this design opens up a broad range of potential applications, including gesture recognition and physiological monitoring. Future research could delve into more advanced biomimetic designs and explore the integration of advanced materials and fabrication techniques to continue advancing the performance and versatility of flexible sensors. In addition, a thorough exploration of the tensile, torsional, and interlayer shear stresses that act on the sensor will contribute to expanding the application scope of flexible pressure sensors.

## Data Availability

Data are available upon request from the authors.

## Supplementary Materials

The following supporting information can be downloaded at: https://www.mdpi.com/article/10.3390/s25082415/s1, Figure S1. Change curve of the maximum electrode plate displacement under different pressures. Figure S2. Unidirectional microstructure could cause shift of sensor surface according to the simulation results. Figure S3. The hysteresis characteristic curve of the sensor. Figure S4. The durability test curve of the sensor. Table S1. Comparison of sensor properties [18,24,25,26,27,28,29,30].
