# Supplementary material for: Human Skin-Inspired Staggered Microstructures for Optimizing Sensitivity of Flexible Pressure Sensor
Source: Sensors (Basel). 2025 Apr 11;25(8):2415. doi: 10.3390/s25082415 (PMC12030931; doi:10.3390/s25082415)
Supplement: Supplementary file 1 [file sensors-25-02415-s001.zip › sensors-3563541-supplementary.pdf]

# Supporting Information

**Human Skin-Inspired Staggered Microstructures for Optimizing Sensitivity of Flexible Pressure Sensor**

*Kechen Li and Yuanyuan Yang **

School of Aerospace Engineering, Xiamen University, Xiamen 361000, China

*E-mail: yangyuanyuan@xmu.edu.cn

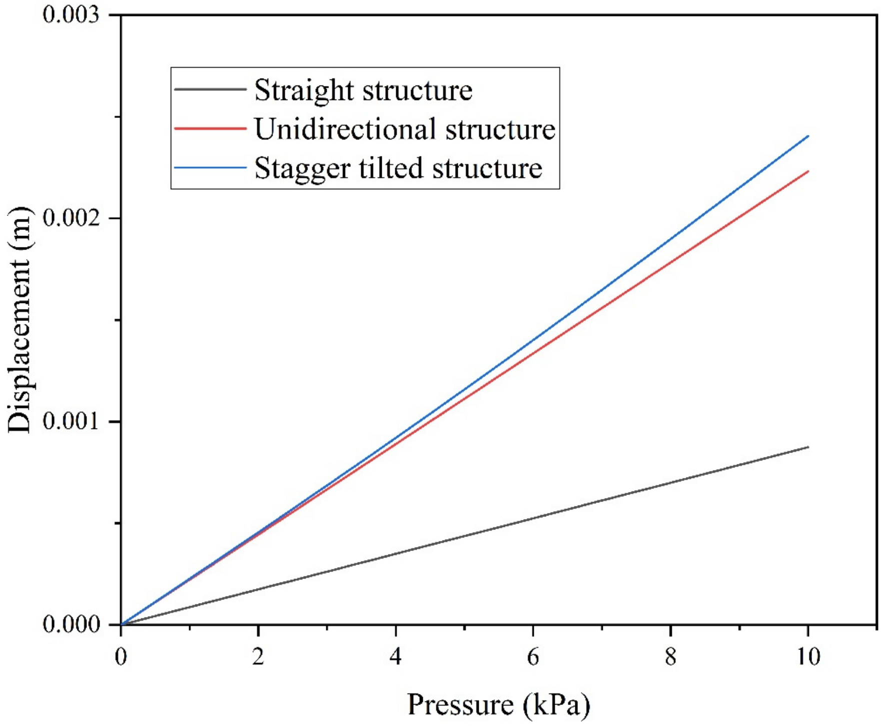


**Figure S1**. Change curve of the maximum electrode plate displacement under different pressures.

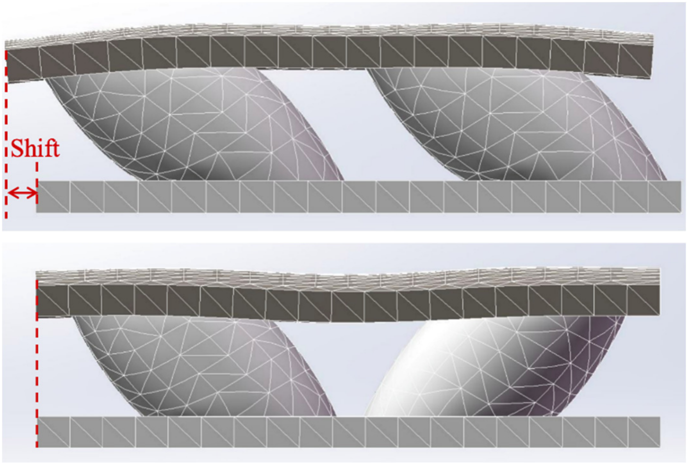


**Figure S2**. Unidirectional microstructure could cause shift of sensor surface according to the simulation results.

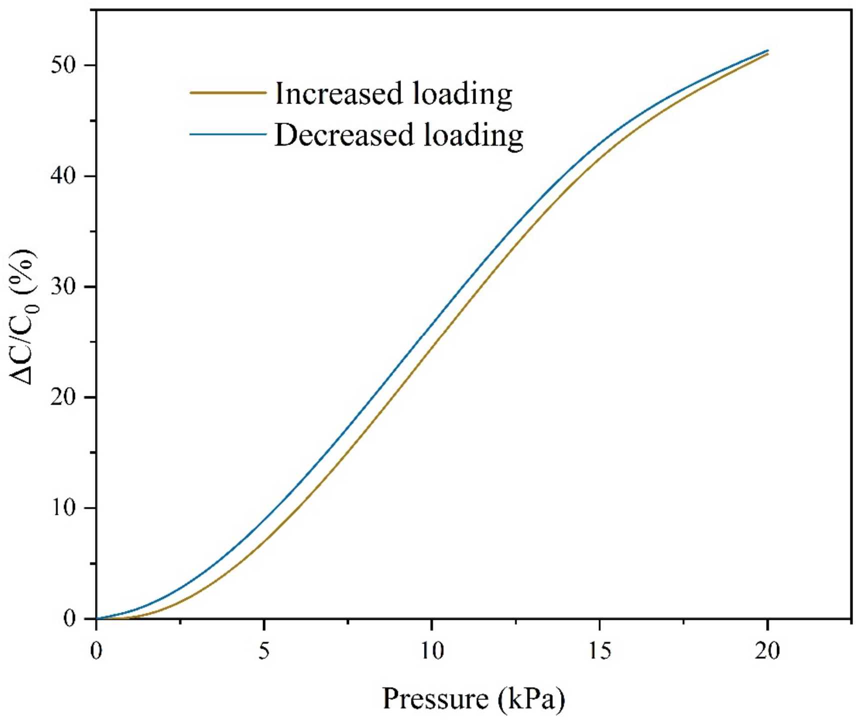


**Figure S3**. The hysteresis characteristic curve of the sensor.

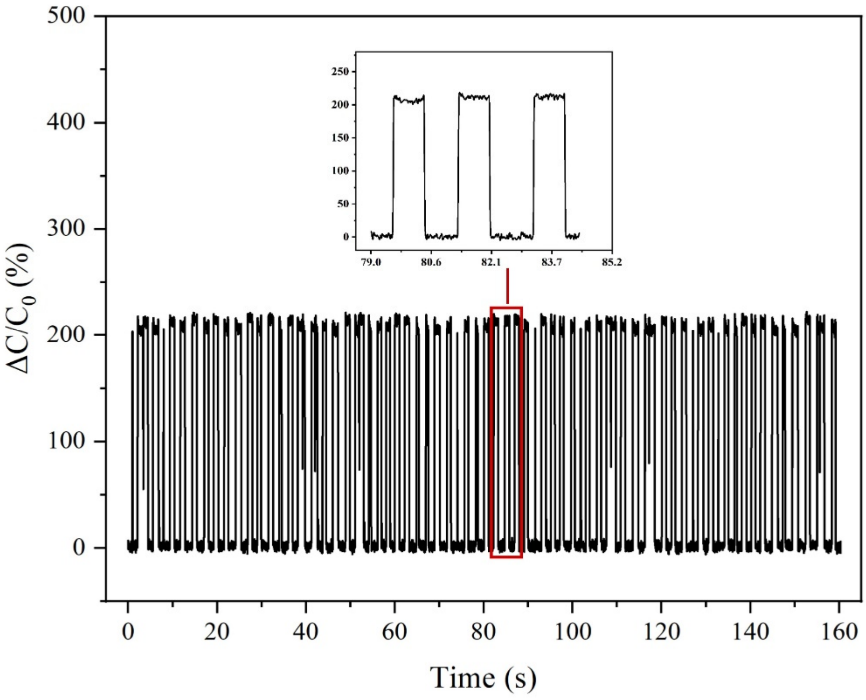


**Figure S4**. The durability test curve of the sensor.

**Table S1**. Comparison of sensor properties.

| Ref. | Mechanism. | Materials. | Structure. | Response time. | Sensitivity. |
|---|---|---|---|---|---|
| [1] | Piezoresistive | PDMS/MWCNTs | Microporous | 203 ms | 0.0179 $kPa^{-1}$ |
| [2] | Piezoresistive | PVA/glycerol/ PGA | - | 230 ms | GF = 2.14 |
| [3] | Piezoresistive | CNT/GNP/PDMS | Two-dimensional | 620 ms | GF = 18.49 |
| [4] | Piezoresistive | Graphene/MXene-PDMS | Porous skeleton | 110 ms | 0.053 $kPa^{-1}$ |
| [5] | Capacitive | PDMS | Slant pyramid | - | 0.0281 $kPa^{-1}$ |
| [6] | Capacitive | PDMS | Micro dome | 225 ms | 0.0044 $kPa^{-1}$ |
| [7] | Piezoresistive | GNP/SR/AgNF | Interlocked truncated sawtooth | 200 ms | 0.45 $V N^{-1}$ |
| [8] | Piezoresistive | Graphene/Silver composites | Interlaced bump | ~350 ms | 0.034 $kPa^{-1}$ |
| Our work | Capacitive | PDMS | Staggered slant cylinder | ~0.2-0.3s | 0.071 $kPa^{-1}$ |

## Reference

[1] Jiang, R.-Y. Lv, Y.-L. Zou, and H.-L. Peng, "Flexible pressure sensor with wide pressure range based on 3D microporous PDMS/MWCNTs for human motion detection," Microelectronic Engineering, vol. 283, p. 112105, 2024/01/01/ 2024, doi: https://doi.org/10.1016/j.mee.2023.112105.

[2] C. Hu, Y. Zhang, X. Wang, L. Xing, L. Shi, and R. Ran, "Stable, Strain-Sensitive Conductive Hydrogel with Antifreezing Capability, Remoldability, and Reusability," ACS Applied Materials & Interfaces, vol. 10, no. 50, pp. 44000-44010, 2018/12/19 2018, doi: 10.1021/acsami.8b15287.

[3] Y. An et al., "A carbon nanotube/graphene nanoplatelet pressure sensor prepared by combining 3D printing and freeze-drying method," Journal of Polymer Research, vol. 31, no. 5, p. 129, 2024/04/16 2024, doi: 10.1007/s10965-024-03972-y.

[4] S. Sang, Z. Jing, Y. Cheng, C. Ji, Q. Zhang, and X. Dong, "Graphene and MXene-based Sponge Pressure Sensor Array for Rectal Model Pressure Detection," Macromolecular Materials and Engineering, vol. 306, no. 10, p. 2100251, 2021/10/01 2021, doi: https://doi.org/10.1002/mame.202100251.

[5] S. Li, X. Cui, and Y. Yang, "Oblique Pyramid Microstructure-Patterned Flexible Sensors for Pressure and Visual Temperature Sensing," ACS Applied Materials & Interfaces, vol. 15, no. 51, pp. 59760-59767, 2023/12/27 2023, doi: 10.1021/acsami.3c12625.

[6] E. Thouti et al., "Flexible capacitive pressure sensors using microdome like structured polydimethylsiloxane dielectric layers," Sensors and Actuators A: Physical, vol. 335, p. 113393, 2022/03/01/ 2022, doi: https://doi.org/10.1016/j.sna.2022.113393.

[7] Wang, Y., Zhu, L., Mei, D., & Zhu, W. (2019). A highly flexible tactile sensor with an interlocked truncated sawtooth structure based on stretchable graphene/silver/silicone rubber composites. Journal of Materials Chemistry C, 7(28), 8669-8679. doi:10.1039/C9TC02356A

[8] Zhu, L.; Wang, Y.; Mei, D.; Jiang, C. Development of Fully Flexible Tactile Pressure Sensor with Bilayer Interlaced Bumps for Robotic Grasping Applications. Micromachines 2020, 11, 770. https://doi.org/10.3390/mi11080770
